# On the Inhalation of Ether in Labour

**Published:** 1847-10

**Authors:** Jonathan Clark

**Affiliations:** Lower Merion, Near Philadelphia


*On the Inhalation of Ether in Labour.* By JONATHAN CLARK, M. D., of Lower Merion, near Philadelphia.

To the Editor of the Medical Examiner.

DEAR SIR,—I send you an account of some obstetric cases, in which the inhalation of Ether was used with not unpleasant results.

CASE 1.—July 19th, at 10 o'clock, P. M., I saw Mrs. ——, aged 18, in labour with her first child. An examination shewed the head to be presenting, with the vertex to the left sacro-iliac junction, membranes entire, and the os uteri dilated to the extent of two inches.

She had been in labour since 5 o'clock; the pains had not been very severe, but were increasing in force and frequency, occurring at intervals of three minutes, the os uteri being moderately rigid, and external parts quite so.

The progress of labour was scarcely perceptible, and there appeared to be an unusual want of fortitude and power of control over her feelings, on the part of my patient. As there appeared to be nothing to contraindicate it, I resolved to use the ether. In two minutes from the commencement of the inhalation, she pushed away the sponge and exclaimed, I am losing my senses. On being assured that it would relieve her pain and do her no injury, she renewed the inhalation, and was fully under its influence in five minutes. From the time I first saw her, her pulse had been 80, but upon the application of the ether it fell to 75, and continued at that rate during the labour.

She appeared to be almost unconscious of pain, judging from her actions, but the expulsive efforts of the uterus were not quite so frequent as at first, but more efficient. She said but little except to ask for more, when the ether had evaporated from the sponge. On one occasion she exclaimed, "more, more, I am suffering dreadfully." This was during a pain. She would hold the sponge wet with the ether, to her mouth and nostrils, and inhale with eagerness, till her power of supporting it was lost, when her hand would fall, and with it the sponge, in this way guarding effectually against an over-dose. After remaining quiet a few minutes, she would upon the recurrence of the pain, apply the sponge and inhale again as eagerly as before.

In one hour and forty minutes from the first use of the ether, she gave birth to a male child, quite vigorous and noisy. I then took away the sponge before making an effort to remove the placenta, which came away in a few minutes, the uterus contracting promptly. For ten minutes she continued to insist upon having more of the ether, and not till she had recovered her senses fully, did she seem willing to give it up. It was fully ten minutes after the birth of the child, when she inquired whether it was born, having no recollection of any thing that had occured since inhaling the vapour.

Convalescence was rapid, without any unpleasant symptom, both mother and child doing well.

CASE 2d. July 31st, I was called to see Mrs. O., of Blockley, aged 28 years, in labour with her third child. At 11, P. M., when I saw her, she had been in pain for several hours. The pains were not frequent nor of much force. On examination, I found the head presenting, with the vertex to the left acetabulum, the os uteri dilated to the extent of about three inches, and the membranes entire. I then left her for two hours; and when I returned, the pains had increased in force and frequency; the membranes had been ruptured, though the labour had made little progress.

Although the case was not such as I should have preferred for the use of the ether, I decided on using it. Her general health had been very poor, her digestion much impaired, and a variety of nervous symptoms also rendered her quite miserable.

After inhaling the vapour for a few seconds, the usual cough troubled her somewhat. This however soon subsided, and in seven minutes she dropped the sponge, being entirely under its influence. The pains were increasing in force, and partly roused her from her lethargy, when she would reapply the sponge and again obtain relief. At a quarter of 3, A. M., she gave birth to a very large male child without any consciousness of suffering. The placenta came away almost immediately, no flooding followed, nor any other unpleasant symptom, except a sense of uneasiness in the head, and lightness on raising it. The mother and child have done and are doing well.

CASE 3. August 3d, at 4 o'clock, P. M., I saw Mrs. D. of Blockley, aged 28, in labour with her fifth child. She had been in labour two hours when I saw her. The pains were quite severe, and the labour had made considerable progress. The os uteri was well dilated, the membranes had ruptured, and the head was presenting with the vertex to the left acetabulum.

As she was suffering very much from the violence of the pains, I thought proper to relieve her by using the ether.

She made no objection to its use, and in three minutes from the time of commencing the inhalation, was fully under its influence. Her pulse was 88, when she commenced inhaling, and it fell to 70. It afterward rose to 78.

She seemed to be conscious of her relief from pain, which was evinced by the eagerness with which she seized the sponge, and inhaled again, when the effects of a former inhalation had partially passed away.

In forty-five minutes after first inhaling the ether, she was delivered of a female child of average size, and quite vigorous. The placenta came away in a few minutes; but not till the effects of the ether had passed off. She complained somewhat when it came away, being conscious of pain at the time; but of all that had occurred, and of all her pains, or more properly speaking, of all the expulsive efforts of the uterus, from the time she first inhaled the vapour till after the child was born, she had no recollection whatever. Nothing unpleasant occurred during convalescence.

Case 4th. Mrs. N., of Blockley, aged 26, was taken unwell on the afternoon of Saturday, the 14th September. She complained of great weakness, vertigo, sickness of stomach, and pains in the back and limbs. The bowels were disturbed several times during the afternoon and evening; the evacuations were black and very fetid The matter ejected from the stomach, as she vomited repeatedly, was also of a bilious character. Her constitution had been very much impaired three years since, by an attack of autumnal fever, from the effects of which she has not yet recovered.

During this her first pregnancy, she has suffered much from indigestion, a train of nervous symptoms of a distressing character, together with general *œdema.*

At 8 o'clock in the morning, after a restless night, of which she has no recollection, she was seized with convulsions, which continued to recur ;—the interval between the convulsions decreasing, while the violence and duration of the paroxysms increased. At 10 o'clock, when I saw her, she had had six convulsions.

Her perceptive faculties were altogether obliterated. As each paroxysm subsided, she was observed to recover her faculties less perfectly, till they were wholly lost, with the exception, perhaps, of the ability to feel pain, which appeared to be regained simultaneously with the power of the uterus to contract ; a slight contraction of which served only to usher in another paroxysm. An examination showed that but little progress had been made in the labour; the os uteri was dilated to the extent of an inch and a half, the membranes were entire, and the head was presenting. Her pulse was one hundred and ten in the minute. As she had complained of head ache before the convulsions came on, and as the pulse appeared of a character to bear it, I took sixteen ounces of blood from the arm.

This had no favourable effect. On the contrary, the spasms continued to increase in force and frequency. The pulse rose to one hundred and thirty-five in a minute, becoming much weaker. The extremities became cold, notwithstanding the application of sinapisms, and the surface was generally cold and clammy, and of a livid hue. Under these circumstances, it occurred to me that the vapour of ether might act as a stimulant, and also change the disordered action then existing. By administering the vapour, a worse state of things could not be induced than already existed, for it was evident to me, from the untoward progress the case had made, that a few more convulsions would destroy her.

All hope of a favourable result was lost, inasmuch as the labour made no perceptible progress. The lethargy succeeding a paroxysm of convulsions was accompanied by a want of contractile power in the uterus, and as soon as this was in a measure regained, and the uterus began to contract, another paroxysm would occur, preventing the further progress of the labour.

Under these circumstances I thought that if I could substitute the lethargy from the inhalation of ether, for the existing one, there would be a great point gained; *the one putting an entire stop to the labour, the other having no such effect.*

During the interval between each paroxysm, I hadexamined the state of the os uteri, hoping to find it dilated sufficiently to enable me to introduce my hand for the purpose of turning, but this was not the case, as there was very little dilatation. In a few minutes after the ninth paroxysm had passed off, I applied a sponge, well moistened with ether, over the mouth and nostrils The patient soon began to rub her nose violently, pushing away the sponge as soon as it was reapplied, till she was prevented by holding her hands. Her countenance in a minute or two lost its deathly hue, and resumed a more natural appearance. In less than ten minutes the whole surface became warm, and much more natural.

The pulse fell to one hundred and twenty-five; the interval between the paroxysms increased more than one-half, and their duration, when they did recur, was much lessened. Uterine contractions now ceased to have their former effect of bringing on the convulsions, *so that I could observe several distinct and efficient pains or contractions between the paroxysms.* The os uteri, as a consequence, began to dilate, but not as yet sufficiently to admit of the introduction of the hand.

I did not venture to apply the sponge long enough to produce a complete lethargy, but removed it when her opposition to its application in a measure ceased. I was fearful, if a complete state of lethargy was induced in her then low condition, she might not react. After being three hours and a half under the influence of the ether, the uterus was sufficiently dilatable to admit of the gradual introduction of the hand, the membranes which were still entire, were ruptured, and I succeeded in obtaining one foot, which was brought down and secured with a tape. Owing to the ungovernable restlessness of my patient, and to the powerful contractions of the uterus, I had great difficulty in finding the other; and when I had succeeded in getting it partly down, it offered so much resistance to my efforts, that I was apprehensive it might not be a fellow to the one I had. After comparing the direction of the toes, I ventured to exert a little more force, and brought away the child.

It was still living, though much exhausted, the lungs required inflation before it breathed, but after respiration was once established it did very well. The placenta came away promptly, and there was no flooding.

No vapour was given after the child was delivered. The mother still continued in a stupor, with convulsions at intervals of forty-five minutes, till 4 o'clock next morning, when they ceased. She took, during the night, as an antispasmodic, forty drops of tr. assafœtida in milk, at intervals of two hours. In the evening her pulse was one hundred and twenty-eight, and quite feeble. I should mention that the convulsions had diminished in force, and continued to do so till they ceased.

At eight in the morning, the stupor still continuing, she took ten grains of calomel, and in one hour a tea-spoonful of fluid ext. of senna, which was repeated every hour for four hours, when it operated on the bowels, producing copious black and very fetid evacuations. From this time she recovered rapidly: the day following she noticed some things and answered questions. Her tongue had been sadly bitten; she could not account for its soreness; has no recollection of any thing that has occurred, and thinks it strange that her child could have been born without her knowledge. In two weeks she was about her room, having convalesced rapidly without an unpleasant symptom. The child, a fine boy, is doing well.

Case 5th. September 4th, at 3 o'clock, A. M., I was called to see Mrs. ——, of Lower Merion, in labour with her third child. She had been complaining all night. Her pains, when I first saw her, were not so frequent, nor so severe as they had been for some hours previous, and they continued to decline till they passed off altogether for a time during the middle of the day.

They again came on in the afternoon with more force and increased frequency. An examination shewed that no progress whatever had been made in the labour. The usual vaginal secretion was wanting, the vagina being preternaturally dry and rigid. On inquiry I found that there had been a leucorrhœa of some standing. As there had been no progress, I left her, and returned at 7 o'clock. An examination now shewed the labour to be progressing rapidly; the os uteri was well dilated, the head presenting with the vertex to the left acetabulum. The membranes were not ruptured, and the secretion of mucus quite abundant. I ruptured the membranes, after which the pains became very severe. I now suggested the use of the ether to my patient and her friends, to which they immediately consented. In a minute after the application of the sponge, she began to talk in an excited strain on subjects unconnected with her case, till a pain came on, when she spoke of it in a light and merry mood, repeating the words, "a pain! a pain!" rapidly and mincing her words in such a way as to convey the idea that she was suffering very little. During the intervals between the pains, she talked wildly and incessantly, even when the sponge was applied to her face.

Her pulse was eighty-five when she commenced taking the vapour; it fell to seventy-six, and after she ceased to inhale, it rose to ninety-six. She seemed conscious of relief from the inhalation, and would call for more gas as she termed it, inhaling it with eagerness. She frequently remarked that she knew perfectly well what she was doing, knew her friends who were present, although she would call for them as though they were out of her sight, asking where they were when they were standing immediately over or by her. She boasted that the gas could not affect her: that her mind was superior to its influence. As the labour approached its termination, she seemed to suffer acutely, screaming violently, asserting that she would die,—"all your gas wont save me!"

At five minutes to 9 o'clock, P. M., she gave birth to a large male child, much larger than either of her others had been. She appeared to be perfectly conscious of its birth, but remarked, although she had suffered so much and complained so bitterly, that she could not have lived through it, if it had not been for the gas. This she repeated several times, after she had ceased to inhale the vapour, and after its effects had passed off.

The placenta came away in eight minutes after the birth of the child. There were no unpleasant symptoms, and the convalescence was rapid. A slight degree of inflammation of the child's eyes, which shewed itself in a few minutes after its birth, and which was attributable to the leucorrhœa of the mother, yielded to a mild astringent wash.

Case 6th. September 11th, at 4 o'clock, A. M., I saw Mrs. E., of Roxborough, aged 37, in labour with her sixth child. She had been ill all the preceding day, but the pain had not been very severe till night, when the paroxysms became quite regular and continued so till I saw her. Her pulse was 84 to the minute. On making an examination, I found the head well down in the pelvis, the os uteri dilated to the extent of several inches, and the vertex presenting to the left acetabulum.

The membranes were entire; I ruptured them, and as she was suffering acutely, though there was not a prospect of its continuing long, I proposed to her the use of the ether, and she gladly embraced the proposition.

In less than two minutes she was completely under its influence, and in a lethargic state, the pulse falling to 72. She was partially roused from this by the recurrence of a pain, but the re-application of the sponge re-induced the lethargy, during the continuance of which, and without any further inhalation, she gave birth to a healthy and vigorous female child. She knew nothing whatever of its birth, and was delighted with the relief she had experienced, saying she had been in a dream from the time she took the vapour. The mother and child are doing well.

